# Disorder of intracellular cobalamin metabolism: Importance of rapid diagnostic illustrated by a case report of early-onset methylmalonic aciduria and homocystinuria, cobalamin C type

**DOI:** 10.1016/j.heliyon.2025.e42086

**Published:** 2025-01-23

**Authors:** Etienne Mondesert, Bastien Baud, Agathe Roubertie, Jean-François Benoist, Pierre-Edouard Grillet, Jean-Paul Cristol, Marie Céline Francois-Heude, Manuel Schiff, Stéphanie Badiou

**Affiliations:** a Department of Biochemistry, University Hospital of Montpellier, Montpellier, France; b Institute for Neurosciences of Montpellier, University of Montpellier, INSERM U 1298, Montpellier, France; c Department of Pediatric Neurology, University Hospital of Montpellier, Montpellier, France; d Department of Biochemistry, Assistance Publique-Hôpitaux de Paris, Paris, France; e Université Paris-Saclay, Paris, France; f PhyMedExp, Université de Montpellier (UM), Inserm, CNRS, Montpellier, France; g Reference Center for Inborn Errors of Metabolism, Department of Pediatrics, Necker Hospital, Assistance Publique-Hôpitaux de Paris, Université Paris cité, Paris, France

**Keywords:** Methylmalonic aciduria and homocystinuria, Cobalamin C type, Inborn error of metabolism, Homocysteine, Methylmalonic acid, Vitamin B12, Case report

## Abstract

Methylmalonic aciduria and homocystinuria, cobalamin C type (cblC), constitute the most common inborn error of intracellular cobalamin metabolism. Here, we report the case of a 6-month-old child, presenting severe subacute neurological decline associated with failure to thrive. Biochemical tests indicated a disorder of intracellular cobalamin metabolism, with elevated urinary and plasma methylmalonic acid levels associated with high plasma homocysteine concentrations, with normal plasma vitamin B12 concentrations. Diagnosis was later confirmed by genetic analysis which identified two pathogenic variants on the *MMACHC* gene: c.271dupA (p.Arg91lysfs∗14) paternal allele and c.388T > C (p.Tyr130His) maternal allele. The patient responded well to hydroxocobalamin treatment, with a rapid recovery of symptoms and a normal growth at 2.8 years of follow-up. This case illustrates the importance of early diagnosis of cobalamin metabolism disorders by prescribing adequate biochemical tests.

## Highlights

• Cobalamin metabolism disorders can be quickly suspected with adequate biochemical tests.
• Hydroxocobalamin supplementation allowed rapid and dramatic recovery of symptoms.
• *MMACHC:*c388T > C mutation have been associated with early and late-onset forms of disease.

## 1 Introduction

Inborn errors of cobalamin (Cbl) metabolism are a group of disorders caused by enzymatic defects in the Cbl processing pathway. There are nine number of complementation groups, each of which is attributed to the mutation of a different gene and are named Cbl-A to Cbl-X types (Supplementary Fig. 1, [1]). Cbl under the form methyl-Cbl acts as cofactor for the cytoplasmic methionine synthase to transform homocysteine in methionine in the remethylation pathway. The inborn errors of Cbl metabolism named CblD-HC (MIM number: 277410), CblE (MIM number: 236270) and CblG (MIM number: 250940) types lead to isolated increase in homocysteine (i.e. isolated remethylation defects). In the mitochondria, the form adenosyl-Cbl, acts as cofactor for the methylmalonyl-CoA mutase (MMUT) to form succinyl-CoA from methylmalonylCoA. CblD-MMA (MIM number: 277410), CblA (MIM number: 251100) and CblB (MIM number: 251110) variants lead to isolated elevation of methylmalonic acid that should be distinguished from MMUT deficiency (MIM number: 251000). CblC (MIM number: 277400), CblD-Combined (MIM number: 277410), CblF (MIM number: 277380) and CblJ (MIM number: 614857) types alter the cytoplasmic and mitochondrial Cbl process, resulting in both elevation of homocysteine and methylmalonic acid. Some specific symptoms can be present for certain inborn errors of Cbl metabolism, but the main signs are mostly identified during the first year of life and can include growth retardation, neurological impairment, eating disorder, most often associated with megaloblastic bone marrow failure [2].

Methylmalonic aciduria and homocystinuria, cobalamin C (CblC) type is the most common inborn error of Cbl metabolism with an incidence ranging from 1/100 000 in New York state [3] to 1/60 000 in California [4]. The gene responsible for the disease was identified in 2005 as the *MMACHC* gene (1p34.1) [5]. At that time, 250 cases were known and in 204 individuals, the 271dupA pathogenic variant had a prevalence of 40 % of all disease alleles [5] but was found to be up to 55 % in European population [6]. In 2019, more than 500 cases were reported with early-onset disease (before age 12 months), representing 89 % of the cases [7]. Recently, compound heterozygous mutations in *PDRX1* gene associated with heterozygous *MMACHC* mutations have been associated with presentation comparable to CblC type deficiency [8]. The pathogenic variants lead to a loss of function or reduced activity of the protein product, disrupting the formation of methyl-Cbl and adenosyl-Cbl, which are required for the methionine synthase and the MMUT activity respectively.

Clinical symptoms cover a wide range of manifestations due to two main forms of the disease: early-onset or late-onset. In early-onset disease, growth retardation accompanied by feeding difficulties are the most common first clinical signs. However, the entire organism can be impacted with neurological involvement (seizures, acute encephalopathy, brain atrophy and white matter disease), renal (atypical hemolytic uremic syndrome), cardiovascular (various structural heart defect potentially leading to severe heart failure [9]), and haematological manifestations. Craniofacial dysmorphia, including long face, high forehead, flat philtrum, can be also encountered in approximatively 20 % of patients [10]. In the late-onset form, clinical presentations are less acute, more heterogeneous. In addition to microangiopathic renal or pulmonary disease, macrocytic anemia, stroke and thrombosis, they can also include behavioral or psychiatric issues, as well as insidious cognitive impairment and other neurological manifestations [[11], [12], [13]]. Lifelong hydroxocobalamin (OHcbl) supplementation is necessary to treat symptoms and prevent complications. Most of the patients are however affected by progressive retinal disease with various degrees of severity from subtle retinal nerve fiber layer loss to advanced macular and optic atrophy [14].

Here we report a case of an early-onset methylmalonic aciduria and homocystinuria, CblC type, presenting with a neurological impairment with growth retardation, illustrating the importance of further metabolic investigations.

## 2 Admission in the emergency department

**Fig. 1:**
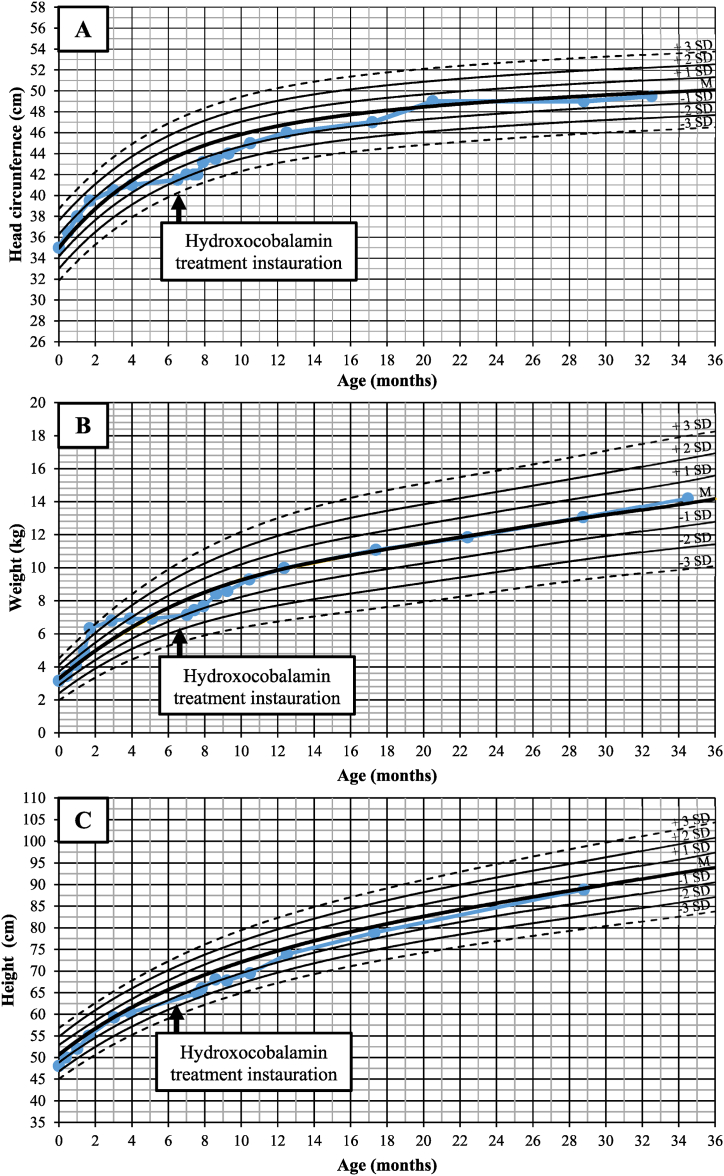
A. Head circumference evolution. B. Weight evolution. C. Height evolution. SD: standard deviation, M: median.

A 6-month and 2-weeks-old male infant was referred for psychomotor regression in the last 2 months and failure to thrive. At admission, patient's weight was 6.2 kg (−2.1 standard deviation (SD)) with no weight gain for the last 3 months, height was 64 cm (−1.8 SD) and head circumference stagnated around 41.5 cm (−1.5 SD) since age 4 months (0 DS) (Fig. 1). The child was uncomfortable, irritable, with continuous moaning. Permanent tremors of all four limbs and opisthotonos were noticed. Clinical examination disclosed poor eye contact, no head control, pyramidal tract signs with brisk tendon reflexes, axial hypotonia contrasting with limb hypertonia.

The patient was the first child of healthy unrelated parents with balanced nutritional intakes; benign coronavirus-19 infection occurred at 7 months of gestation, pregnancy and delivery were otherwise uneventful with normal birth parameters (weight: 3.1 kg/-0.3 SD, height: 48 cm/-1.5 SD, head circumference: 35 cm/0 SD) at 38 weeks of gestation. Early psychomotor development was normal: good eye contact and dedicated smiles from the first weeks, ability to hold the head at 2 months, ability to grab objects and emit babbling at 4.4 months. The patient was exclusively breastfed from birth, and introduction of nutritionally-adequate complementary solid foods from 5 months (mashed vegetables and fruits). He exhibited an episode of rhinitis at age 4 months, and a viral gastroenteritis one month later. After this episode at 5 months, the parents reported progressive feeding difficulties especially with solid foods, leading to a dramatic decrease of daily caloric intakes (at admission: 40 kcal/kg/day). Although the clinical and neurodevelopmental examination were considered normal at the 4 and 5 months visits, the parents reported a progressive loss of motor and communicative skills and neurological symptoms since the end of the 4th month.

On admission arterial blood gas pH, lactate, electrolytes and ammonium measurments were normal. Complete blood count with differential revealed a normocytic, normochromic anemia (Table 1).

**Table 1:** Biological parameters at admission.

**Parameter**	**Value**	**Unit**	**Reference ranges**
**Arterial blood gas**			
pH	7.43		7.35–7.45
PCO2	36	mmHg	27–41
PO2	53	mmHg	83–108
O2 saturation	89	%	94–98
Bicarbonates	24	mmol/L	22.0–26.0
Base excess	−0.1	mmol/L	−2.0–2.0
**Plasma biochemistry**			
Glucose	5.4	mmol/L	3.3–5.6
Urea	4	mmol/L	1.3–5.8
Creatinine	35	μmol/L	15–32
Uric acid	365	μmol/L	88–370
Sodium	140	mmol/L	136–145
Potassium	4.9	mmol/L	3.5–5.7
Chlore	104	mmol/L	97–106
Bicarbonates	20	mmol/L	22–29
Anion gap	21	mmol/L	
Proteins	66	g/L	43–69
Albumin	46	g/L	38–54
CRP	8.5	mg/L	<5.0
Lactate	1.7	mmol/L	0.5–2.2
Amoniaemia	39	μmol/L	<50
**Whole blood haematology parameters**			
Hemoglobin	89	g/L	100–140
Hematocrit	27.5	%	30–42
Erythrocytes	3.26	T/L	3.4–5.0
Mean corpuscular volume	84	fL	70–100
Mean corpuscular hemoglobin concentration	27.3	pg/L	22–35
Thrombocytes	538	G/L	150–400
White blood cells	8.24	G/L	5.0–20.0

## 3 Hospitalization in the department of paediatric neurology

Breastfeeding intakes were supplemented by tube feeding using formula to reach 93 kcal/kg/day (dietary proteins were stopped for 48 hours until an aminoacidopathy or organic acidemia was ruled out). After blood sampling, L-carnitine 320 mg/d, and polyvitamin supplementation (B1 100 mg/day, B8 10 mg/day, B2 50 mg/day, B9 10 mg/day, mg/day, B6 180 mg/day and OHcbl 0.16 mg/kg/day) were provided orally. Given the irritability and rigidity, the patient received oral clonazepam and melatonin.

**Fig. 2:**
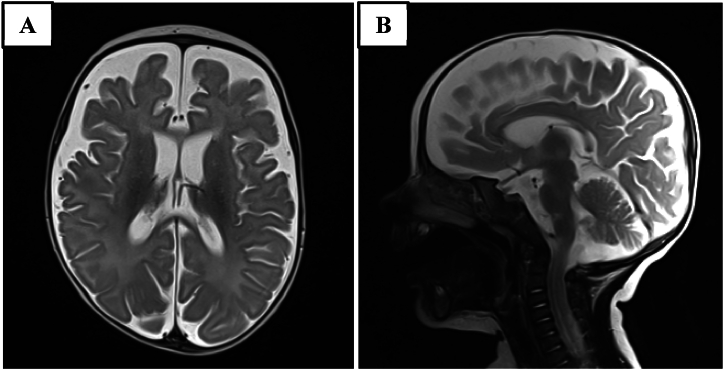
Brain MRI at admission. A. T2 TSE axial sequence. B. T2 TSE sagittal sequence.

Brain MRI (Fig. 2) showed cerebral atrophy with enlargement of the subdural spaces, mostly in the bilateral fronto-temporal region, filiform appearance of corpus callosum and brainstem biometry below 3rd percentile. EEG recording disclosed slow background rhythms associated with slow spikes waves. Optic funduscopy, brainstem visual evoked potentials, cardiac and renal ultrasound scan were normal.

Metabolic investigations including lipids, vitamin B12, folates, pyruvate, lactate, copper, ceruloplasmin, very long chain fatty acids and pipecolic were within normal ranges (Table 2). Besides anemia, a B12 metabolism disorder was suggested by an abnormal acylcarnitine profile, associating normal free carnitine to very high propionylcarnitine (27 μmol/L, N:<1) and methylmalonylcarnitine (0.9 μmol/L,N:<0.1). In parallel, there was a massive increase in total homocysteine concentration (256 μmol/L, N:<9). Amino acid profile revealed a low methionine concentration (6 μmol/L, N:12–48) pointing towards a remethylation defect. Methylmalonic acid measurements in plasma and urine were in favor of a Cbl metabolism disorder, with values of 246 μmol/l (N < 0.5) and 948 mmol/mol creatinine (N < 5), respectively.

**Table 2:** Metabolic investigations.

**Parameter**	**Value**	**Unit**	**Reference ranges**
**Plasma**			
Total cholesterol	3.43	mmol/L	2.36–5.32
Triglycerides	1.26	mmol/L	0.59–2.63
Vitamin B12	779	pmol/L	145–569
Folates	77.7	nmol/L	8.83–60.8
Lactate	2.1	mmol/L	0.5–2.2
Pyruvate	117	μmol/L	40–170
Copper	20.1	μmol/L	14.9–18.9
Ceruloplasmin	0.28	g/L	0.16–0.37
Pipecolic	1.2	μmol/L	<5
C22:0	48	μmol/L	28–100
C24:0	36	μmol/L	20–85
C26:0	0.28	μmol/L	0.3–1.2
Homocysteine	**256**	μmol/L	2.0–9.0
Methylmalonic acid	**246**	μmol/L	<0.5
**Urine**			
Methylmalonic acid	**978**	mmol/mol creat	<5
**Plasma amino acid**			
Amino alpha butyric acid	15	μmol/L	10–40
Glutamic acid	85	μmol/L	36–136
Alanine	277	μmol/L	180–495
Arginine	75	μmol/L	23–105
Citrulline	16	μmol/L	10–48
Cystine	9	μmol/L	15–52
Glutamine	788	μmol/L	350–800
Glycine	199	μmol/L	150–400
Hydroxyproline	26	μmol/L	0–40
Isoleucine	48	μmol/L	38–122
Leucine	81	μmol/L	84–236
Lysine	128	μmol/L	100–294
Methionine	**6**	μmol/L	12–48
Ornithine	82	μmol/L	32–160
Phenylalanine	52	μmol/L	36–120
Proline	244	μmol/L	90–330
Sarcosine	0	μmol/L	<1
Serine	190	μmol/L	80–235
Tyrosine	60	μmol/L	33–110
Valine	207	μmol/L	137–340

**Fig. 3:**
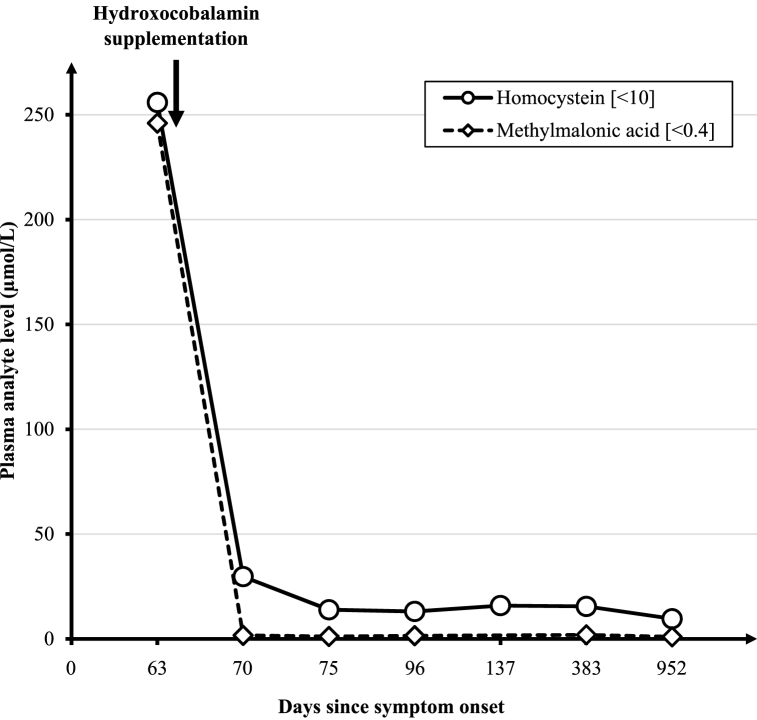
Methylmalonic acid and homocystein plasma levels (μmol/L) evolution since symptom onset. Normal ranges (μmol/L) of the two analytes are indicated. The introduction of hydroxocobalamin supplementation is also represented.

Taken together, the association of normal vitamin B12 plasma concentration with severe hyperhomocysteinemia and methylmalonic acid increase were indicative of an intracellular metabolism of vitamin B12 disorder, corresponding to the group CblC, CblD-Combined, CblF or CblJ types. Following these results, oral OHcbl was switched for intramuscular injections (1 mg 3 times a week corresponding to 0.07 mg/kg/d and one week after oral supplementation introduction), in addition to oral anhydrous betaine (250 mg/kg/d), oral L-carnitine (50 mg/kg/d), oral L-methionine (50 mg/d) and oral folinic acid (5 mg/week). The other vitamins, clonazepam and melatonin were discontinued. A couple of days later, the patient showed a dramatic clinical improvement with good eye contact, weaning from the gastric tube, and 47 g/day weight gain. After one week of treatment, no anemia was observed, total homocysteine plasma concentrations decreased at 29.7 μmol/L associated to a decrease of plasma methylmalonic acid to 1.7 μmol/L (Fig. 3).

## 4 Diagnosis

The diagnosis of methylmalonic aciduria and homocystinuria, CblC type was confirmed on a next generation sequencing panel of 216 genes responsible for metabolic defects and hepatic insufficiencies. Two pathogenic variants on the *MMACHC* (NM_015506) gene were identified; c.271dup (p.Arg91lysfs∗14) and c.388T > C (p.Tyr130His). Segregation analysis showed that c.271dup was inherited from the father and c.388T > C from the mother. The two variants were previously associated with the disease, c.271dup accounting for 40–55 % of mutations in European or American subjects was classified as pathologic according to the American college of medical genetics and genomics (ACMG) classification. c388T > C variant was described in four precedent cases [5,6,15,16] and was also classified as pathologic using ACMG classification meeting the following criteria: PM1, PM2, PM3, PM5, PP3 and PP5.

## 5 Follow-up

OHcbl was changed from 1mg triweekly to 2 mg biweekly intramuscularly (0.07–0.05 mg/kg/d); the remaining drugs were maintained at the same dosages according to weight. The patient was able to walk unaided at 18 months of age, he emitted short sentences at 2.3 years. Growth recovery was achieved in a short period of time, as illustrated by head circumference evolution in Fig. 1. At last examination (2 years 10 months) clinical examination was normal, weight was 14.2 kg (0 SD), height 89 cm (0 SD) and head circumference 49.5 cm (−0.4 SD). Formal neuropsychological evaluation was normal as well as eye fundoscopy. Total plasma homocysteine was 9.6 μmol/L, plasma methionine 64 μmol/L and methylmalonic acid 1 μmol/L (Fig. 3).

## 6 Discussion

This case-report describes an early-onset methylmalonic aciduria and homocystinuria, cblC type presentation through neurological manifestations and failure to thrive. As an inborn error of metabolism with endogenous toxic accumulation, clinical manifestations could occur several months after birth although very early onset severe presentations can occur in the first weeks after birth. Considering the favourable evolution under treatment, it appears mandatory to perform metabolic investigations to have the earliest diagnosis in every suspicion of inborn error of metabolism [7].

The patient was not diagnosed through NBS, as cblC disease is not included in the newborn screening program in France. NBS is performed in numerous countries, using the proprionylcarnitine to acetylcarnitine (C3/C2) ratio. This could have important impacts on patient evolution, as recent large cohort studies have shown that hydroxocobalamin supplementation before onset was associated with better prognosis [17,18]. Moreover, it has recently been shown that high-dose OHcbl therapy (0,55 mg/kg/d) as well as early therapy after newborn screening (NBS) allowed improvement of neurodevelopmental test results compare to standard dose (0,09 mg/kg/d) [19]. Some cases have also reported the potential of early and high-dose OHcbl treatment in pregnant woman carrying children affected by inborn errors of intracellular Cbl metabolism such as CblE or CblC [20,21].

The patient inherited one mutation from each parent, the first one (c.271dupA) is the most common mutation in western world patients living with methylmalonic aciduria and homocystinuria, cblC type. However, the second mutation (c.388T > C, p.Tyr130His) was only found in four previous cases [5,6,15,16]. One case concerned a 34-year-old Italian woman, p.Tyr130His/p.Tyr222∗, describing a late pregnancy loss, placenta showing severe vascularization abnormalities, which can be observed in preeclampsia and the patient seemed to present signs since she was 20 years old, with an impaired renal function (thrombotic microangiopathic with lesions mainly in the glomerulus). The second described case was about a 13.6-year-old male with the same genotype as the present case, presenting an atypical hemolytic uremic syndrome (HUS). The two last cases only displayed genotypes c.388T > C + c.481C > T and c.388T > C + c.471G > C and ages, 17-year-old female, 1 to 4-year-old child respectively.

Comparing the patient reported herein to the only one literature case having the same genotype [15], we can observe differences in the symptoms and age of diagnosis, suggesting a variable expressivity of the mutation. The previous patient had severe hypertension and acute renal failure without any reported neurological impairment. In contrast, the present patient had an early onset phenotype without HUS and with neuro-hematological impairments, suggesting that neurological symptoms are not linked directly with the *MMACHC* variants. Moreover, it is still unclear if neurological impairment is caused by structural neurodevelopmental abnormalities or B12 deficiency. Nevertheless, the clinical significance of the c.388T > C variant requires further functional analysis. Regardless, the dramatic improvement with relatively low OHcbl dosages and normalization of the biochemical parameters suggests this variant may result in a milder phenotype than most of the typical early-onset presentations. Furthermore, another missense variant on the same amino acid (c.389A > G, p.Tyr130Cys) was reported in early-onset presentation [22], as well as late-onset atypical HUS and neuropsychiatric deficits cases [[23], [24], [25]].

Methylmalonic aciduria and homocystinuria CblC, CblD-Combined, CblF and CblJ types are mainly responsive to intramuscular OHcbl administration [2,13]. Recently, it has been shown that the addition of betaine at 250 mg/kg/d could benefit CblC patients throught increasing methionine and S-adenosyl methionine concentrations [26]. In the present case, a biological and clinical response was achieved a week after treatment initiation. Sustained treatment response is generally obtained with normal growth [13], as observed here at the last visit. A therapeutic perspective could be an implantable subcutaneous device coupled to a portable infusion pump, allowing continuous and higher dosage administration with better compliance from the patient and parents, due to the high frequency of intramuscular administrations [27,28].

Other specific presentations exist such as renal impairment, including atypical hemolytic uremic syndrome [15]. Overall, cardiac, vascular and haematological manifestations could occur. In cblC type, special attention should be paid to ophthalmologic evaluation as maculopathy are described [2,14]. Presentations of late-onset diseases are more heterogeneous, with peripheral and central neuropathies, macrocytic anaemia, stroke, thrombosis and behavioral or psychiatric symptoms [13].

In summary, an early-onset methylmalonic aciduria and homocystinuria, cblC type was evidenced through metabolic investigations of a neurological impairment associated with failure to thrive. Diagnosis of inborn error of vitamin B12 metabolism was performed within three days after hospitalization, highlighting the importance of inborn error of metabolism screening if clinical and neuroimaging are suspicious. Thanks to this rapid diagnosis orientation, the introduction of an adapted treatment with intramuscular OHcbl could be considered and allowed a favourable evolution, before diagnosis confirmation which was subsequently provided by genetic analysis.

## CRediT authorship contribution statement

**Etienne Mondesert:** Writing – original draft, Formal analysis, Data curation. **Bastien Baud:** Writing – original draft, Formal analysis, Data curation. **Agathe Roubertie:** Writing – review & editing, Resources, Investigation. **Jean-François Benoist:** Writing – review & editing, Resources. **Pierre-Edouard Grillet:** Writing – review & editing. **Jean-Paul Cristol:** Writing – review & editing. **Marie Céline Francois-Heude:** Writing – review & editing, Investigation. **Manuel Schiff:** Writing – review & editing, Investigation. **Stéphanie Badiou:** Writing – review & editing, Supervision, Conceptualization.

## Ethics and consent statement

Written informed consent was obtained from the patient parents for the publication of this case-report.

## Funding

This research received no specific grant from funding agencies in the public, commercial, or not-for-profit sectors.

## Declaration of competing interest

The authors declare that they have no known competing financial interests or personal relationships that could have appeared to influence the work reported in this paper.
