# Supplementary material for: Disorder of intracellular cobalamin metabolism: Importance of rapid diagnostic illustrated by a case report of early-onset methylmalonic aciduria and homocystinuria, cobalamin C type
Source: Heliyon. 2025 Jan 23;11(3):e42086. doi: 10.1016/j.heliyon.2025.e42086 (PMC11795796; doi:10.1016/j.heliyon.2025.e42086)
Supplement: Multimedia component 1 [file mmc1.docx]

*Lysosome*

*OH-cbl (III)*

*OH-cbl (III)*

***cblC/cblX***


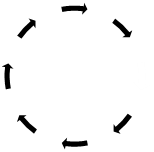


*Succinyl*

*-CoA*

*MCM-Ado-cbl*

*Methyl*

*malonyl-CoA*

***Krebs***

***cycle***

*Methyl*

*Malonic acid*

*Mitochondria*

*Cytoplasm*

*Cell membrane*

**Associated genes:**

**cblA**: *MMAA*

**cblB**: *MMAB*

**cblC**: *MMACHC /*

*PDRX1*

**cblD**: *MMADHC*

**cblE**: *MTRR*

**cblF**: *LMBRD1*

**cblG**: *MTR*

**cblJ**: *ABCD4*

**cblX:** *HCFC1*

***cblA***

***cblF/cblJ***

*Transcobalamin II*

*receptor*

*Transcobalamin II*

*OH-cbl (III)*

*cbl (II)*

*Ado-cbl*

*cblC-cbl (II)*

*MS-cbl (II)*

*Homocysteine*

*Methionine*

***cblD-***

***Combined***

***cblG***

***cblE***

***cblB***

***cblD-MMA***

***cblD-HC***

*cbl (II)*

*cbl (II)*

*MS-Me-cbl*

**Figure 1.** Impact of cobalamin variants (cblA to cblX) proteins on intracellular metabolism of vitamin B12. Names of the gene associated with cobalamin variants are detailed. Ado-cbl: adenosyl-cobalamin, cbl: cobalamin, OH-cbl: hydroxycobalamin, me-cobalamin: methyl-cobalamin, MCM : methylmalonyl-CoA mutase, MS: methionine synthase.
